# Transcriptional regulation: efficient genetic engineering tools for non-conventional yeasts

**DOI:** 10.1093/femsyr/foag032

**Published:** 2026-08-03

**Authors:** Shabana Haneef, Yongjin J Zhou, Fan Bai

**Affiliations:** Division of Biotechnology, Dalian Institute of Chemical Physics, Chinese Academy of Sciences, Dalian 116023, PR China; University of Chinese Academy of Sciences, Beijing 100049, PR China; Division of Biotechnology, Dalian Institute of Chemical Physics, Chinese Academy of Sciences, Dalian 116023, PR China; Dalian Key Laboratory of Energy Biotechnology, Dalian Institute of Chemical Physics, Chinese Academy of Sciences, Dalian 116023, PR China; Division of Biotechnology, Dalian Institute of Chemical Physics, Chinese Academy of Sciences, Dalian 116023, PR China; Dalian Key Laboratory of Energy Biotechnology, Dalian Institute of Chemical Physics, Chinese Academy of Sciences, Dalian 116023, PR China

**Keywords:** non-conventional yeasts, transcriptional regulation, dynamic regulation, CRISPR/Cas regulation, genetic engineering tools

## Abstract

Non-conventional yeasts are recognized as valuable hosts for producing biofuels, pharmaceuticals, and other high-value chemicals, owing to their diverse physiological traits, ability to utilize various substrates, and greater tolerance to environmental stresses compared to conventional model yeast *Saccharomyces cerevisiae*. To fully optimizing metabolic flux toward desired products, effective genetic engineering tools enabling precise modulation of gene expression and coordinated control of metabolic pathways are essential. In this context, we discussed classical transcriptional regulation tools like promoters, and transcription factors, alongside innovations in synthetic biology that allow metabolic engineering in non-conventional yeasts to produce higher biofuels and other useful products, promoting the development of sustainable resources, and assisting the development of innovative bio-products. It also discussed innovative programmable technologies, such as CRISPR/Cas-mediated transcriptional activation and repression, as well as dynamic regulatory systems that can fine-tune metabolic routes and balance cellular resources. Strategies for promoter engineering, transcription factor manipulation for transcriptional regulation, and metabolic rewiring were highlight as methods to boost pathway efficiency and yields. This review concluded with current challenges and future directions, focusing on integrating synthetic biology and systems biology to create robust, controllable transcriptional frameworks for next-generation yeast cell factories.

## Teaser

This review concluded with current transcript regulation genetic engineering tools, such as promoters, transcription factors, dynamic, and CRISPR/Cas-mediated transcriptional regulation systems, focusing on integrating synthetic biology and systems biology to create robust, controllable transcriptional frameworks for non-conventional yeast cell factories.

## Introduction

The current climate crisis and fossil fuel depletion demand new approaches to biofuel and biochemical production. Yeasts have influenced human history for centuries by enabling the production of essential metabolites such as ethanol, acetic acid, and lactic acid through natural fermentation (Maicas 2020, Voidarou et al. 2020). These compounds have formed the foundation of the food and beverage industries. Moreover, yeasts have facilitated the sustainable production of biofuels and complex natural products (Moon et al. 2016, Ehrenworth and Peralta-Yahya 2017). These products include monoterpene indole alkaloids (MIAs), benzylisoquinoline alkaloids (BIAs), hormones, neurotransmitters, vitamin B12, and strictosidine (Nielsen and Keasling 2016, Lian et al. 2018, Chen et al. 2020) which are challenging to extract from natural sources but can now be produced microbially. Several examples of successful heterogeneous biosynthesis of mammalian and plant natural products (NPs) like hydrocortisone (Kelly and Kelly 2003), artemisinic acid (Peplow 2016), resveratrol (Li et al. 2015), strictosidine (Brown et al. 2015), and (S)-reticuline (Pyne et al. 2020) are also produced using yeasts. Yeasts have evolved robust regulatory networks to maintain metabolic homeostasis, yet achieving industrially relevant titer, rate, and yield (TRY) for natural product (NP) biosynthesis requires extensive transcriptional and metabolic rewiring (Lian et al. 2018). Despite these advances, industrial-scale biosynthesis is constrained by pathway complexity and metabolic burden (Basan et al. 2020, Courdavault et al. 2020).

Recent advances in metabolic engineering have expanded the use of non-conventional yeasts (NCY), particularly in *Komagataella phaffii* (Cai et al. 2022, Shrivastava et al. 2023), *Yarrowia lipolytica* (Blazeck et al. 2014, Zhang et al. 2023), and *Ogataea polymorpha* (Yu et al. 2023, Xie et al. 2024), which offer distinct physiological advantages and industrial robustness. Non-conventional yeasts exhibit superior stress tolerance, metabolic diversity, and distinctive growth characteristics, which render them valuable for specialized industrial applications (Ciani et al. 2016). Moreover, these yeasts address challenges associated with conventional yeasts, including hyper-glycosylation and allergenic residues (Stöckmann et al. 2009) and demonstrate resilience to heat, acid, and toxins (Wang et al. 2025). Despite these advantages, their industrial application remains constrained by the limited availability of advanced genetic tools, resulting in cellular stress and inefficiency during heterologous pathway expression (Inokuma et al. 2020). These challenges underscore efficient genetic regulation tools (promoters, terminators, gene editing) as a central bottleneck and key engineering layer for scalable bioproduction. In promoter engineering, upstream DNA sequences regulate transcription through interactions with RNA polymerase and transcription factors (Yan et al. 2022a).

**Figure 1:**
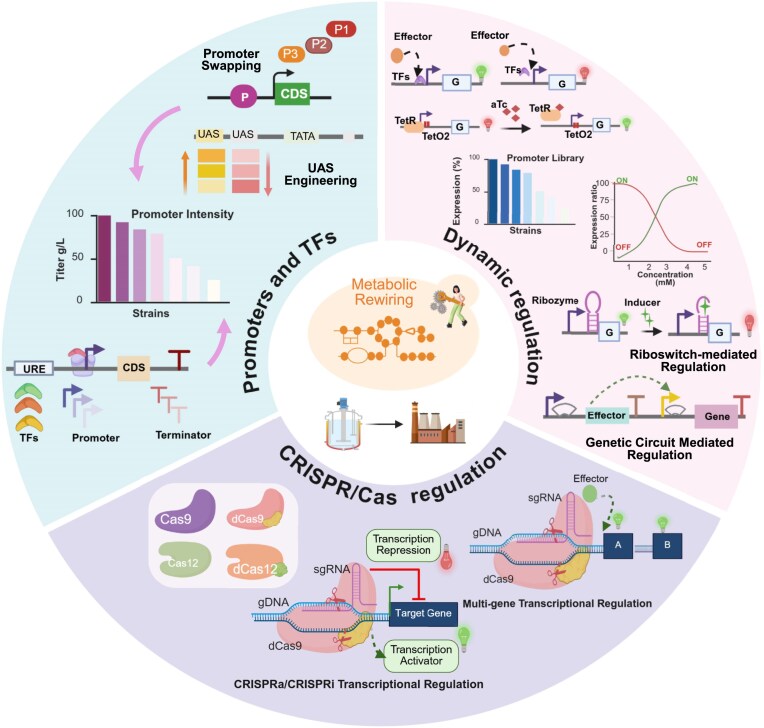
Metabolic engineering necessitates the accurate construction of pathways for the biosynthesis of natural products. This involves the intricate engineering of non-conventional yeast cell factories facilitate industrial-scale production. Both the development of metabolic pathways and bioengineering require precise optimization of gene expression. Among the various strategies employed, promoter engineering is the most widely used approach, including techniques like promoter tuning, regulation of UAS core promoters or hybrid promoters, transcription factor-mediated promoter regulation, and inducible dynamic gene expression regulation. Additionally, CRISPR/Cas-based genome editing enhances gene expression regulation and is a vital tool for advancing metabolic engineering. Image created with BioRender.com.

In this review, we evaluate recent advances in transcriptional regulatory tools and their transformative roles in non-conventional yeast biotechnology. Recent progress in native and hybrid promoter engineering, transcription factor-based strategies, dynamic regulatory systems, which include metabolite-responsive and exogenous inducible modules such as the Tet system, RNA-level approaches like riboswitches and CRISPR/Cas9-mediated transcriptional regulation, has enabled precise gene expression control in industrially relevant non-conventional yeasts. The emerging applications of these tools for optimized bioproduction emphasize that transcriptional fine-tuning expands the metabolic capabilities of these organisms (Fig. 1). Future directions include integrating multilayered regulatory networks and data-driven design to engineer robust, high-performing non-conventional yeast strains for sustainable biomanufacturing.

### Transcription regulation in metabolic engineering

The vigorous developmental achievements in the field of metabolic engineering along with synthetic biology, have already accomplished the heterologous production of microbial cell factories for bulk chemical compounds and pharmaceutical products (Zhang et al. 2019, Luo et al. 2021). The central dogma in molecular biology, which posits a flow of biological information initiated by transcription and translation, along with the regulatory network, has gained widespread attention in the field of metabolic engineering. The conventional approach for the production of these cell factories for the microbial system is the genetic regulation of the metabolic pathway, either by upregulation, downregulation, and knock out (Liu et al. 2013). Though the metabolic pathway’s complexity often disturbs the expected phenotypes, like upregulation causing gene overexpression to a toxic level, and downregulation/knockout affecting cell growth due to deficiencies in metabolites (Ghogare et al. 2020). Moreover, the single gene manipulation disrupts the cell homeostasis for normal cellular processes like growth, division, or metabolic stress (Xu et al. 2020). In recent years, significant progress has been made in developing transcriptional regulatory tools in these yeasts, including the identification and engineering of native and synthetic promoters, transcription factors (TFs), riboswitch-based regulatory elements, and CRISPR-mediated transcriptional control systems. These tools have enabled improved and tunable control of gene expression, facilitating efficient pathway balancing and enhanced product yields.

### Transcriptional regulation by promoters

Transcriptional regulation is central to metabolic engineering, with promoters controlling mRNA synthesis and stability. Functional and structural aspects of native and synthetic yeast promoters have been extensively reviewed (Hubmann et al. 2014), and computational tools now facilitate rational promoter selection for synthetic circuits (Tyo et al. 2011). Promoters play a central role in regulating the spatial, temporal, and quantitative aspects of gene expression, thereby critically influencing metabolic and cellular processes (Ji et al. 2024). The selection of suitable promoters, rather than defaulting to the strongest available, is essential in NCY’s engineering to prevent cellular toxicity and metabolic burden (Rebello et al. 2018). Table 1 summarizes general native promoters from various non-conventional yeasts. Constitutive endogenous promoters such as P*_ADH_*, P*_GAP_*, P*_TEF1_*, and P*_GCW14_* enable strong gene expression, whereas inducible promoters responsive to metabolites, including methanol utilization promoters (P*_AOX1_*, P*_AOX2_*, P*_DAS2_*, P*_MOX_*, P*_EYEK_*) and rhamnose-dependent P*_LRA3_*/P*_LRA4_*, enable optimized production. P*_TEF1_* frequently employed for robust expression in *Y. lipolytica* (Gu et al. 2023), and a library of 81 endogenous promoters has been classified by strength as strong, medium, and weak to support high-level production, demonstrated by the highest reported titer (95.64 mg/l) of salidroside (Wang et al. 2023). Vogl et al. (2016), obtained various promoters based on carbon sources (methanol and glucose) in *K. phaffii*. However, methanol-induced promoters are the best choice for robust gene expression, producing a 3-hydroxypropionic acid (3-HP) titer of 48 g/l (Wu et al. 2023). Though robust gene expression systems remain particularly important for non-model microorganisms (Riley and Guss 2021), where balanced transcription is essential to minimize metabolic burden (Mao et al. 2024). Promoter size is a critical factor, as large endogenous promoters can hinder the assembly of multi-gene pathways. This challenge has led to the development of compact synthetic promoters (130 bp) to improve the modularity and tunability of complex metabolic pathways (Xu et al. 2021). The demands of metabolic engineering call for the best possible combination of promoters to balance production and growth. As core transcriptional regulators, promoters define circuit architecture and enable the separation of growth and production phases through their broad dynamic range and programmability (Tominaga et al. 2024). Therefore, extensive promoter engineering is essential for the non-conventional yeast cell factories.

**Table 1:** List of general native, engineered, and hybrid promoters in non-conventional yeasts.

Category	Promoter	Yeast species	Application	Reference
Constitutive promoters	P*_GAP_* P*_GAPDH_* P*_TEF1_* P*_ADH3_* P*_ADH1_*	*K. phaffii* *O. polymorpha* *Y. lipolytica*	Glycolytic promoter is widely used for heterologous gene expression; Translation elongation factor promoter widely used for metabolic engineering; Intermediate constitutive promoter.	Ahn et al. 2007, Karaoglan et al. 2016, Massahi and Çalık 2018, Xu et al. 2018, Lin et al. 2024
	P*_GCW14_*	*K. phaffii* *O. polymorpha*	Verified strong expression using *GFP* and product formation.	Liang et al. 2013 Yan et al. 2022a
	P*_PDC_*	*K. phaffii*	Pyruvate decarboxylase: a strong constitutive promoter.	Massahi and Çalık 2018
	P*_PGM_* P*_GLK_* P*_TRI_* P*_GPI_*	*O. polymorpha*	Phosphoglycerate mutase promoter; Glucokinase promoter; Triose phosphate isomerase promoter; Glucose-6-phosphate isomerase promoter.	Yan et al. 2022a
	P*_FBA1_*	*O. polymorpha*	Metabolic pathway expression	Yan et al. 2022a
Inducible promoters	P*_AOX1_* P*_DAS1_* P*_DAS2_* P*_FLD1_* P*_CAT1_*	*K. phaffii*	Methanol-based metabolic engineering; Methanol-free expression systems; Recombinant enzyme production; Stress-inducible promoter.	Vogl and Glieder 2013, Vogl et al. 2018, Nong et al. 2020, Ebner et al. 2025
	P*_XPR2_* P*_EYK1_*	*Y. lipolytica*	Lipid metabolism engineering; Tunable gene expression.	Blazeck et al. 2014
	P*_ICL1_* P*_LRA3_* P*_LRA4_* P*_Mal_*-P*_Per_*	*O. polymorpha*	Inducible (ethanol) responsive.	Yan et al. 2022a
Engineered promoters	P*_AOX1_* variants	*K. phaffii*	Methanol-independent expression.	Prielhofer et al. 2013
	P*_GTH1_* variants	*K. phaffii*	Tunable expression strength.	Flores-Villegas et al. 2023
	P*_GAP_* promoter library		Expression optimization.	Qin et al. 2011, Bai et al. 2025
	P*_GCW14_* library		Methanol-independent diverse range of inducible to target complex metabolic pathways.	Haneef et al. 2026
	PU12, PU13, PC48	*Y. lipolytica*	Fine metabolic tuning; High-titer chemical production.	Kang et al. 2025
Hybrid promoters	UAS1B-TEF	*Y. lipolytica*	Strong gene expression.	Blazeck et al. 2011
	UAS1XPR2-derived promoters	*Y. lipolytica*	Lipid production pathways.	Larroude et al. 2018
	UAS1EYK1		Synthetic biology circuits.	Trassaert et al. 2017
	UAS-based promoters	*O. polymorpha*	Fine metabolic tuning with enhanced production.	Ye et al. 2024
	UAS-based promoters	*K. phaffii*	Increased transcriptional activity for enhanced production.	Ergün et al. 2020 Chen et al. 2025

**Figure 2:**
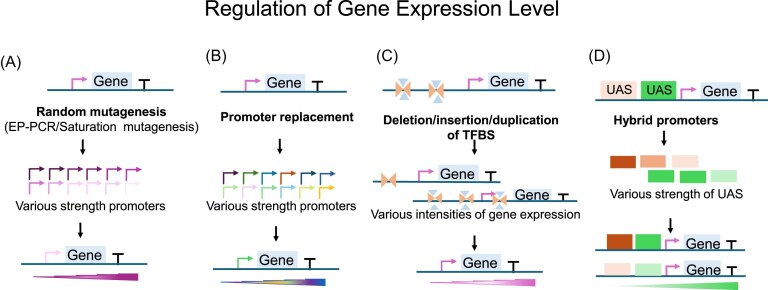
Strategies for gene expression regulation via promoter engineering. Promoter engineering provides a versatile approach for fine-tuning transcriptional activity and optimizing gene expression levels in microbial cell factories. Several strategies can be employed to generate promoters with different strengths and regulatory properties. (A) Random mutagenesis, including error-prone PCR (EP-PCR) and saturation mutagenesis, introduces nucleotide variations into promoter regions to create promoter libraries with a wide range of transcriptional strengths. (B) Promoter replacement involves substituting native promoters with promoters of varying strengths to modulate target gene expression. (C) Modification of transcription factor binding sites (TFBSs) through deletion, insertion, duplication, or rearrangement alters transcription factor interactions and enables graded control of promoter activity. (D) Hybrid promoter engineering combines upstream activation sequences (UASs) with core promoters, or incorporates multiple UAS elements, to generate synthetic promoters with enhanced or tunable transcriptional outputs. Collectively, these promoter engineering approaches enable precise regulation of gene expression, facilitating pathway balancing, metabolic flux optimization, dynamic control, and improved production of desired metabolites and recombinant proteins.

Promoter engineering strategies, including mutagenesis of regulatory elements, combinatorial promoter, and modification of transcription factor (TF) binding sites (TFBS), enable graded control of transcriptional strength and environmental responsiveness (Ito et al. 2013, Curran et al. 2014, Horie et al. 2020) (Fig. 2). Although these approaches modulate promoter strength and dynamic range, TF-ligand specificity generally remains unchanged, underscoring the value of orthogonal TF systems for programmable gene circuits (Cautereels et al. 2024). The integration of synthetic TFs and CRISPR-based transcriptional regulators further enables precise and reversible gene modulation (Yan et al. 2022a). The yeast Tunable Expression Systems Toolkit (yTEST) expands inducible control using small-molecule-responsive promoters (O’Laughlin et al. 2023), while hybrid promoters combining upstream activating sequences with defined core elements provide enhanced and context-dependent expression profiles (Blazeck et al. 2014, Shabbir et al. 2015, Teng et al. 2024).

These strategies have directly improved metabolic performance for non-conventional yeast factories (Fig. 2). For instance, tandem amplification of upstream activation sequences (UAS) elements in *Y. lipolytica* generated a tunable promoter library with a 400-fold transcriptional range. This hybrid promoter library was constructed by placing multiple tandem UAS1B activating sequences upstream of either the minimal LEU2 promoter, with 1 to 32 UAS1B repeats, or TEF promoter fragments of various lengths fused to 8 or 16 UAS1B repeats. Separating the activation module from the core promoter allowed precise control of transcriptional output and produced promoters with significantly enhanced expression strength (Blazeck et al. 2011). Based on the same strategy, a combination of bacterial regulatory factors (FadR) with P*_TEF1_* in *Y. lipolytica* developed a fatty acid-sensitive hybrid promoter, which can produce 160 mg/l of *ω*-hydroxy palmitic acid (Park et al. 2021). A combinatorial approach for promoter engineering in *K. phaffii* generated a diverse range of promoters based on the core P*_AOX1_*. This not only helps to describe the genetic architecture of P*_AOX1_* clearly but also improves the promoter strength (Portela et al. 2018). In a separate study, fusion of the bacterial regulatory proteins with the yeast, along with a linker protein to the core promoter of the P*_AOX1_*, results in 126 constitutive promoters (cTRLD) and 162 inducible promoters (iTRLD), which have an expression range from 16% to 520% and 30% to 500% of the native promoter, respectively (Zhu et al. 2022). Furthermore, a randomized P*_GAP_* library was constructed by Lai et al. (2023), demonstrating an 18% increase in activity at a monoterpene titer of 1.18 mg/l compared to the native promoter. Our group characterized constitutive and growth-phase-dependent promoters in *O. polymorpha* that enhanced acetyl-CoA flux, thereby enabling *β*-elemene production at 5.24 g/l. Using a transcriptional data-driven promoter engineering strategy, the constitutive and growth phase-dependent promoter library was expanded to include promoters with graded strengths relative to P*_GAP_*. These promoters were incorporated into hybrid growth phase-responsive constructs for precise metabolic control. This approach enabled coordinated downregulation of GAP and phase-dependent expression of biosynthetic modules, thereby redirecting flux toward acetyl-CoA and increasing *β*-elemene production (Ye et al. 2024). In *K. phaffii*, systematic promoter mapping led to the engineered promoter PS2, supporting 3-hydroxypropionic acid titers up to 23 g/l on methanol. They used a rational promoter-engineering strategy to computationally identify the core promoter (BDGP Neural Network Promoter Prediction tool) and predicted the UAS TFBS using Alibaba2 of the endogenous P*_PYC2_* promoter. Stepwise UAS truncation removed inhibitory regulatory regions and defined the minimal functional UAS. The optimized promoter showed significantly increased transcriptional activity. However, tandem UAS duplication and heterologous AOX1-UAS fusion did not further enhance expression, emphasizing the importance of UAS and core promoter compatibility (Chen et al. 2025). Random and saturation mutagenesis of methanol-responsive promoter libraries enabled graded control over production in comparison to the native promoter and improved production (Qin et al. 2011, Portela et al. 2018).

In the context of modern artificial intelligence and machine learning, strategies for promoter engineering have advanced significantly through the use of powerful computational tools. In *K. phaffii*, promoter design utilizing convolutional neural networks (CNN) and high-throughput screening (HTS) enabled the construction of a P*_AOX1_* mutant that is 5.3 times more effective than the native promoter. Furthermore, validation using real-time PD-L1 production demonstrated that this mutant increased the titer by up to 30% (Cheng et al. 2025). This field is rapidly evolving, continually developing new tools and strategies to support more rational and effective promoter design.

### Transcriptional regulation by transcription factors (TFs)

**Figure 3:**
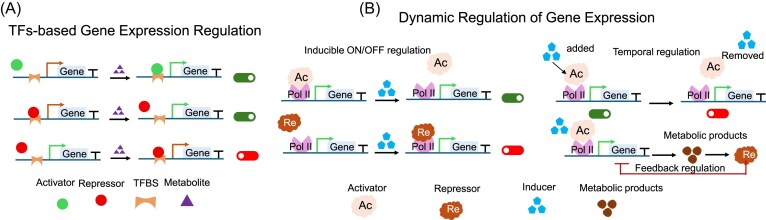
Strategies for gene expression via TFs and dynamic regulation. (A) Transcription factors (TFs)-based gene regulation. Gene expression is controlled through transcription factor binding sites (TFBS) upstream of core promoter elements (e.g. TATA box). (B) Dynamic gene expression control. Inducible genetic circuits enable reversible control of gene expression in response to external stimuli. The system toggles between on and off states depending on inducer availability, allowing dynamic regulation. The binding of TFs modulates RNA polymerase II (Pol II) recruitment, enabling transcriptional activation or repression in response to cellular signals. Temporal regulation enables precise control of transcription by adding or removing inducers at specific times. Feedback regulation uses metabolic products as signals to adjust transcription factor activity, automatically tuning gene expression based on pathway performance and cellular metabolism.

Transcription factor (TF)-based regulation is a key strategy for metabolic engineers working with non-conventional yeasts because it can reprogram entire gene expression patterns through precise, single-point interventions (Andersson and Sandelin 2019). TF engineering enables global transcriptional rewiring, allowing coordination among multiple downstream genes, particularly valuable in non-conventional yeasts whose regulatory landscape diverges from that of *S. cerevisiae* (Sun et al. 2026). Table 2 summarized transcription factors and regulatory engineering of TFs in non-conventional yeasts. In methylotrophic non-conventional yeasts, the methanol utilization pathway and the related TFs are crucial for regulating gene expression (Hartner and Glieder 2006). Engineered transcription factors function as sensors and switches, optimizing pathways and preventing the buildup of toxic intermediates (Fig. 3A). Overexpression of the methanol-induced transcription factor 1(*Mit1*), improved the P*_AOX1_* strength up to 2.2-fold (Haghighi et al. 2022). Erden-Karaoğlan et al. (2022) constructed P*_ADH2_* hybrid promoters, achieving a 2.2-fold increase in activity by replacing the repressor with an activator in *Mxr1*-overexpressing cells. These promoters facilitate xylanase production in *K. phaffii*, yielding 165%–200% of the total.

**Table 2:** Transcription factors and regulatory engineering of TFs in non-conventional yeasts.

Category	Transcription factor	Yeast species	Regulation/function	Target promoters/pathways	Reference
Native	*Mxr1* *Mit1* *Prm1*	*K. phaffii* *O. polymorpha*	Methanol metabolism regulatory TFs	P*_AOX1_* P*_DAS1_* P*_FLD1_*	Vogl and Glieder 2013 Shi et al. 2017
	*Mig1*	*K. phaffii* *O. polymorpha* *Y. lipolytica*	Glucose repression regulator	Glycolytic promoters	Shi et al. 2017 Wang et al. 2017 Ergün et al. 2020
	*Gal4*	*K. phaffii*	Crabtree phenotypic regulation by upregulation of glucose uptake	Glycolytic gene	Ata et al. 2018
	*Cat8*	*K. phaffii* *Y. lipolytica*	Carbon source regulator	Carbon metabolism promoters	Barbay et al. 2021
	*Yas1* *Yas2* *Yas3*	*Y. lipolytica*	Lipid metabolism regulator Alkane-responsive transcription factors	Alkane-inducible promoters ALK promoters	Endoh-Yamagami et al. 2007 Hirakawa et al. 2009
	*Yap* like TFs		Oxidative stress response regulator	Stress response genes	Gorczyca et al. 2023
	*Oaf1*/*Pip2*	*Y. lipolytica*	Activates β-oxidation genes	Peroxisomal genes	Hirakawa et al. 2009
	*Snif1*		lipid metabolism regulation	Manipulated for improved lipid biosynthesis	Seip et al. 2013
	*Hap* complex (*Hap*2/3/4/5)	*Y. lipolytica*	Activates respiratory and TCA cycle genes under low glucose	Overexpression alters energy metabolism distributions	Celińska and Zhou 2025
Engineered	Syn TF expression cassettes	*K. phaffii*	Improves methanol promoter strength and stability	*Mxr1* + *Mit1* co-overexpression	Vogl and Glieder 2013
	CRISPR-based TF modulators	*Y. lipolytica* *K. phaffii*	Targeted activation/repression of metabolic genes without changing coding sequences	CRISPR-based TF modulators (dCas9-VP64, dCasEV)	Löbs et al. 2017 Schwartz et al. 2017
	Hybrid TF regulators	*Y. lipolytica*	Engineered activation domains fused to UAS-binding modules for orthogonal control	Hybrid TF regulators with customizable activation	Blazeck et al. 2013 Blazeck et al. 2012 Wang et al. 2017
	Syn TF + UAS		Synthetic TF designed to bind specific UAS for orthogonal modular control	Synthetic TF + UAS promoter pairs	
	Engineered stress-response TFs		Enhanced stress tolerance and robust production	Modified *Yap1, Hsf1* for stress-response	

In *Y. lipolytica*, a TUNE^YAL^ 56-TFs-based promoter swapping library was constructed with seven different expression ranges. Here, they systematically map TF dosage-phenotype relationships and increased betanin production, whereas *SNF2* repression or *GCN4* overexpression improved thermotolerance (Jiang et al. 2025). Complementing these tools, artificial TFs (aTFs) derived from *Arabidopsis thaliana* DNA-binding domains fused to yeast *GAL4* or plant *EDLL* activation domains were functionally characterized in *K. phaffii*, spanning a 0.1-fold to 9-fold transcriptional range with low basal leakage, establishing a truly orthogonal regulatory layer that operates independently of native TF networks (Naseri et al. 2021). Multi-factor synthetic analog circuits expand cellular computation for enhanced pathway control. Synthetic transcription factors (synTFs) enable orthogonal, tunable, and inducible expression of complex and toxic pathways (Naseri 2023), thereby allowing dynamic self-adaptation in response to environmental signals. Biosensors based on transcription factors sense metabolite levels, adjust gene activity to balanced output, and optimize carbon use while reducing strain from foreign pathways (Naseri and Koffas 2020). These biosensors also facilitate rapid mutant screening and high-throughput optimization, thereby expediting robust cell factory development (Park et al. 2024, Lee et al. 2025)

Besides, the chromatin remodeling also involves the regulation of gene expression. Studies in *K. phaffii* (Gupta and Rangarajan 2022)) showed that transcription factor activity depends on chromatin-remodeling cofactors by examining the functional relationship between *Mxr1* and *Trm1* and the effect of *Gcn5* and *Gal83*. This underscores chromatin-level changes as an extra approach for controlling TF activity and fine-tuning gene expression. What makes the current era in TF-based engineering especially exciting is not only the individual achievements but also the expanding, more advanced toolbox available to researchers. CRISPR-Cas9 has enabled precise TF gene editing, including deletion, promoter swapping, and multi-locus integration, even in hosts that typically resist homologous recombination, especially when combined with homologous end joining (NHEJ)-suppression methods such as *Ku70/80* deletion or inducible *RAD52* overexpression (Löbs et al. 2017, Gao et al. 2021).

### Dynamic transcriptional regulation

Dynamic transcriptional regulation is a cornerstone strategy that enables the temporal, tunable, and reversible control of gene expression necessary to optimize metabolic flux, manage toxic intermediates, and decouple cellular growth from biosynthetic production (Celińska and Zhou 2025, Liu and Qi 2025). The regulatory landscape of *K. phaffii, Y. lipolytica*, and *O. polymorpha* has evolved through three broadly defined dynamic regulatory mechanisms: endogenous nutrient-responsive systems, exogenous orthogonal inducers, and precisely programmable regulation (Fig. 3B). In NCYs, most existing dynamic tools come from nutrient-responsive systems. In *K. phaffii*, the methanol-inducible promoter P*_AOX1_* is dominant to achieve induction-based production (Ohsawa et al. 2018). Its transcriptional activation is governed by a network of zinc-finger transcription factors like *Mxr1, Mit1*, and *Trm1*, which coordinately regulate methanol-utilization pathway genes (P*_DAS_*, P*_FLD_*) ( Prielhofer et al. 2015, Wang et al. 2016). Moreover, rhamnose-inducible promoters (P*_LRA3_* and P*_LRA4_*) were reported in *K. phaffii* (Liu et al. 2016). In *Y. lipolytica*, lipid-and erythritol-responsive promoters such as P*_POX2_* and P*_EYK1_* provide substrate-dependent control (Trassaert et al. 2017). While *O. polymorpha* also uses the methanol induction based on P*_MOX_* and P*_FMD_*, along P*_ICL1_* and P*_LRA3_* that induced by ethanol and L-rhamnose (Yan et al. 2022b, Vidal et al. 2023). By using P*_LRA3_*, homologous recombination was enhanced up to 60% in *O. polymorpha*, resulting in 50% higher fatty alcohol (Ni et al. 2024).

However, despite their extensive application, these native inducible regulations have some fundamental limitations. Their activation intrinsically coupled to substrate uptake, catabolism, and cellular physiological state, rendering them prone to carbon catabolite repression, inducer depletion, metabolic burden, and poor temporal resolution (Liu et al. 2025). Industrial challenges include methanol toxicity *O. polymorpha* and *K. phaffii*, while inconsistent substrates in *Y. lipolytica* (Ma et al. 2020, Vogl et al. 2020), which makes it difficult to scale or tightly control production. This highlights the need for regulatory systems that operate independently of host metabolism. To overcome these constraints, the widely used Tet-On/Tet-Off system, which is based on bacterial TetR-derived transcription factors, regulates synthetic tetO-operator containing promoters in response to tetracycline derivatives (Bertram and Hillen 2008). By using Tet on system, Bai et al constructed *K. phaffii* P*_GAP_* synthetic promoter library to regulate homologous recombination (HR) up to 80% (Bai et al. 2025). In a parallel study, we constructed a methanol-independent Tet-On inducible synthetic P*_GCW14_* library and targeted key genes from the malonyl CoA pathway, resulting in better growth and production with 3.7 g/l of 3-HP (Haneef et al. 2026). Wagner et al. established a highly adaptable, Tet-On system for *Y. lipolytica*, utilizing piggyBac transposons for genomic integration and tight transcriptional control (Wagner et al. 2018). In *O. polymorpha*, dual regulation is achieved by combining Tet-Off along with the auxin-inducible system (AID) to regulate the essential kinases (Maekawa et al. 2022).

Riboswitches are RNA elements that control gene expression by changing shape when they bind to certain molecules. While they are mostly found in prokaryotes, scientists have now created synthetic versions that work in eukaryotic cells (Serganov and Nudler 2013). Synthetic riboswitches that respond to theophylline and tetracycline make it possible to control gene activity and sense metabolism in yeast (Kelvin and Suess 2023). Recent progress in synthetic biology has broadened yeast regulatory tools, now enabling post-transcriptional control through RNA riboswitches and aptamers (Hoetzel et al. 2025). For instance, in *K. phaffii*, researchers have used a tetracycline-activated riboswitch to regulate protein synthesis at the translational stage. Incorporating this RNA aptamer within the 5' untranslated region of a gene allowed them to significantly decrease protein expression when the ligand was added (Vieira et al. 2023). This approach is especially useful for modulating crucial proteins and bioproducts (Lee 2025 et al. 2025). Further increase in the dynamic range of these riboswitch-mediated Boolean logic gates has been implied in yeast (Kelvin et al. 2025). They expand the dual input riboswitch approach, which is the combination of two different ligand-binding domains to achieve the high-efficiency hybrid Boolean gates in both basal expression and dynamic range (Kelvin et al. 2026). Recent advances, like combining riboswitches with CRISPR and improving aptamer design, have made riboswitches even more useful as tools in synthetic biology (Cui et al. 2023).

### CRISPR-mediated transcriptional regulation

The CRISPR/Cas system, adapted from bacterial immunity, enables targeted genetic editing, activation, and interference. Multiplex sgRNA vectors assembled with Golden Gate facilitate simultaneous modification of up to 20 genes, making gene manipulation more efficient. Doxycycline-inducible Cas9 improves editing timing and accuracy (Cao et al. 2016). CRISPR/Cas streamlining genomic editing and facilitates more precise pathway engineering while plasmid-based gene regulation remains a robust tool in metabolic engineering, it encounters challenges, including copy number variation and metabolic burden (Ko et al. 2020). Targeted nucleases enable genome insertion by inducing double-stranded breaks that are subsequently repaired via non-homologous end joining (NHEJ) or homologous recombination (HDR).

CRISPR-Cas systems have significantly advanced genome editing by delivering high precision, efficiency, and cost-effectiveness, enabling the concurrent modification of multiple genes (Doudna and Charpentier 2014, Reis et al. 2019). In contrast to *S. cerevisiae*, non-conventional yeasts exhibit limited homologous recombination efficiency, necessitating innovative strategies for targeted genome engineering (Ji et al. 2020, Strucko et al. 2021a). Endonucleases such as Cas9 and Cas12a (Cpf1) facilitate double-strand breaks at designated loci, thereby enabling base editing, gene knockout, marker-free integration, and multiplexed genomic modifications. Cas9 offers broader target accessibility due to its NGG protospacer adjacent motif (PAM) requirement and extensive experimental validation. In contrast, Cas12a recognizes T-rich PAMs and enables efficient multiplex regulation by self-processing clustered regularly interspaced short palindromic repeat (crRNA) arrays. However, Cas12a is constrained by more restrictive PAM requirements and may show variable activity depending on the target locus (Spasskaya et al. 2021, Shi et al. 2022, Zhang et al. 2022). The incorporation of CRISPR/Cas9 technology has fundamentally reshaped the genetic engineering paradigm for non-conventional yeasts, advancing beyond traditional HDR to enable rapid, marker-free, and multiplexed genetic alterations (Shi et al. 2018, Ganesan et al. 2019). Wang et al. (2018) used CRISPR-Cas9 to insert ten copies of the resveratrol pathway genes into *O. polymorpha*’s rDNA locus, raising resveratrol production about 20-fold compared to single-copy integration. Initial implementation emphasizeds efficient gene disruption and standardized genomic integration, highlighted by toolkits such as EasyCloneYALI for *Y. lipolytic*a (Holkenbrink et al. 2018), while recent developments focus on harnessing CRISPR as an adaptable platform for transcriptional regulation (Shi et al. 2018). In addition to genome editing, the CRISPR/Cas system is used to regulate gene expression.

However, genetic expressions are also regulated by the environmental factors in yeast. Therefore, key regulators for metabolic engineering are transcriptional level regulation by using native and synthetic promoters and terminators. Various exemplary studies are; the manipulation of the pathway by using gene encoding squalene synthase (*ERG9*) for sesquiterpenes synthesis (Scalcinati et al. 2012), synthesis of artemisinic acid in yeast strains by using some enzymes of mevalonate pathway for amorphadiene (artemisinin precursor) and artemisinic acid (Paddon et al. 2013). A recent study used CRISPR interference (CRISPRi) to repress undesirable genes and CRISPR activation (CRISPRa) to amplify desired genes in yeast. This strategy led to a vital increase in the production of valuable natural products, showcasing the extreme potential of CRISPR for bioengineering application (Liao et al. 2022). Advancements in synthetic promoters, in silico selection, and CRISPR-Cas systems empower researchers with unparalleled control over gene expression in yeast. This refined transcriptional regulation opens doors for future bioengineering feats, paving the way for sustainable resources and novel byproducts.

### Transcriptional regulation by CRISPR/dCas in yeast

Fine-tuned and scalable gene modulation is essential for efficient cellular factories (Löbs et al. 2018), and CRISPR/Cas systems now enable both genome editing and programmable gene expression control in non-conventional yeasts (Löbs et al. 2017, Patra et al. 2021). Catalytically inactive Cas9 (dcas9), generated by D10A and H840A mutations in the RuvC and HNH domains, retains DNA-binding capacity while abolishing cleavage activity (Jinek et al. 2012). CRISPR activation (CRISPRa) and interference (CRISPRi) systems, utilizing catalytically inactive Cas (dCas) proteins, facilitate targeted modulation of gene expression in non-conventional yeast species. To downregulate the targeted gene expression, CRISPRi not only obstructs RNA polymerase binding/elongation, but efficacy is further enhanced by the assimilation of TFs (activators/repressors) with dCas9. For example, CRISPRi was developed to enable precise editing of *Mxr1* in *K. phaffii*, thereby regulating methanol-inducible genes (*AOX1, DAS1, DAS2*) (Hou et al. 2020). Moreover, an improved homologous recombination in *Y. lipolytica* (Schwartz et al. 2017), and metabolic pathway engineering in *K. marxianus* (Löbs et al. 2018) and multiplexed sgRNA) approaches have demonstrated increased repression efficiency in *Y. lipolytica* (Löbs et al. 2018, Zhang et al. 2018). An improved CRISPRi system in *K. phaffii* was constructed by fusing dCas9 to native repressor domains and using effective gRNA processing, such as Csy4-mediated cleavage, to achieve strong, multiplexed gene repression of fatty acyl-CoA synthetase genes (*FAA1* and *FAA2*), resulting in up to 85% repression. These advances provide a basis for precise, scalable gene regulation in non-conventional yeast (Qiao et al. 2023).

For CRISPRa, dCas9 combined with transcriptional activators such as VP64 or VPR enables gene upregulation, expanding metabolic capabilities, including cellobiose utilization in *Y. lipolytica* (Schwartz et al. 2017). A dual-purpose CRISPR-Cpf1 platform was developed in *Y. lipolytica*, enabling simultaneous gene disruption and transcriptional regulation, where truncated gRNAs allowed Cpf1-based activation or repression, while full-length gRNAs supported efficient genome editing for metabolic engineering applications (Ramesh et al. 2020). CRISPRi and CRISPRa both enable precise control of gene expression via sgRNA design, aiding metabolic pathway tuning and phenotype optimization. While balancing growth and biosynthesis is challenging, multiplexed CRISPR/Cas regulation shows promise for efficient yeast cell factories.

### Future perspectives

Transcriptional regulation plays a significant role in metabolic engineering, particularly in microbial systems. Synthetic promoters and transcription factors offer unparalleled control over gene expression, enabling the production of distinct chemical compounds and pharmaceutical products. CRISPR-mediated transcriptional regulation has been raised as a powerful tool for precise gene activation or repression in microbial systems. Modified Cas proteins, such as dCas9 and Cas12a, allow for targeted gene editing, offering versatility in metabolic engineering applications. These developments open doors for sustainable biofuel production and novel biotechnological innovations.

However, rational design of these regulatory components remains challenging due to the complexity of gene regulatory networks. In this context, artificial intelligence (AI) and machine learning approaches are emerging as powerful tools for predicting and designing functional regulatory elements (Nguyen et al. 2024). This combinatorial approach in promoter engineering, facilitating precise prediction of promoter activity and advancing the development of customized inducible regulatory systems (Cazier and Blazeck 2021). The integration of AI-assisted design with synthetic promoter libraries, transcription factor engineering, riboswitch-based regulatory systems, and CRISPR/dCas-mediated transcriptional control is expected to accelerate the development of precise and tunable gene expression systems in non-conventional yeasts. Furthermore, combining AI-guided design with high-throughput screening and multi-omics datasets will allow systematic mapping of regulatory networks and facilitate the construction of robust microbial cell factories for the production of high-value biochemicals.

Along with improving regulatory precision, future research needs to focus on how scalable, practical, and economically viable transcriptional regulation systems are for industrial use. Many current inducible systems rely on expensive chemical inducers, complicated genetic designs, or regulatory proteins that put extra strain on cells, which makes them less suitable for large-scale production. To move industrial biotechnology forward, it is important to develop affordable and scalable options like auto-inducible circuits, metabolite-responsive biosensors, quorum-sensing systems, and promoters that respond to growth phases. Combining transcriptional regulation with real-time monitoring and dynamic feedback control can help automate pathway regulation during fermentation, which boosts productivity and lowers costs. Researchers should also assess future systems for how adjustable they are, their regulatory performance, long-term genetic stability, ability to work in industrial settings, compatibility with methanol-free and sustainable feedstocks, and their overall effect on cost-effective biomanufacturing.
